# On the Effects of Process Parameters and Optimization of Interlaminate Bond Strength in 3D Printed ABS/CF-PLA Composite

**DOI:** 10.3390/polym12092155

**Published:** 2020-09-22

**Authors:** Syed Waqar Ahmed, Ghulam Hussain, Khurram Altaf, Sadaqat Ali, Mohammed Alkahtani, Mustufa Haider Abidi, Ayoub Alzabidi

**Affiliations:** 1 Department of Mechanical Engineering, Universiti Teknologi PETRONAS, Perak Darul Ridzuan 32610, Malaysia; syed_19000908@utp.edu.my (S.W.A.); khurram.altaf@utp.edu.my (K.A.); 2 Faculty of Mechanical Engineering, Ghulam Ishaq Khan Institute of Engineering Sciences and Technology, Topi 23640, Pakistan; 3 Department of Mechanical Engineering, National University of Sciences and Technology, Islamabad 44000, Pakistan; sadaqat.ali@smme.nust.edu.pk; 4 Industrial Engineering Department, College of Engineering, King Saud University, Riyadh 11421, Saudi Arabia; moalkahtani@ksu.edu.sa (M.A.); 435107660@student.ksu.edu.sa (A.A.); 5 Advanced Manufacturing Institute, King Saud University, Riyadh 11421, Saudi Arabia; mabidi@ksu.edu.sa

**Keywords:** additive manufacturing, 3D printing, interlaminate bonding, interfacial bond strength, laminates, composite, fused deposition modelling, optimization

## Abstract

The scope of additive manufacturing, particularly fused deposition modelling (FDM), can indeed be explored with the fabrication of multi-material composite laminates using this technology. Laminar composite structures made up of two distinct materials, namely acrylonitrile butadiene styrene (ABS) and carbon fiber reinforced polylactic acid (CF-PLA), were produced using the FDM process. The current study analyzes the effect of various printing parameters on the interfacial bond strength (IFBS) of the ABS/CF-PLA laminar composite by employing response surface methodology. The physical examination of the tested specimens revealed two failure modes, where failure mode 1 possessed high IFBS owing to the phenomenon of material patch transfer. Contrarily, failure mode 2 yielded low IFBS, while no patch transfer was observed. The analysis of variance (ANOVA) revealed that printing parameters were highly interactive in nature. After extensive experimentation, it was revealed that good quality of IFBS is attributed to the medium range of printing speed, high infill density, and low layer height. At the same time, a maximum IFBS of 20.5 MPa was achieved. The study presented an empirical relation between printing parameters and IFBS that can help in forecasting IFBS at any given printing parameters. Finally, the optimized printing conditions were also determined with the aim to maximize IFBS.

## 1. Introduction

Additive manufacturing (AM) techniques carry a substantial role in today’s industrial and technological arena. AM offers the flexibility of producing complex geometries while consuming minimal operational time. AM technology has been adopted by a very wide range of fields such as aerospace, automobile, manufacturing, designing, tissue, and biomedical engineering industries [1,2,3,4,5,6]. Fused deposition modelling (FDM) is one of the most widely used AM technologies owing to its operational flexibility and low cost [7]. FDM creates three-dimensional objects by laying up successive layers of thermoplastic material upon one another. Thermoplastic filament, acting as feedstock material, is pulled through rollers and inserted into the heated extruder. The heated extruder then melts and extrudes the material via an extrusion nozzle onto the pre-heated bed, while following the coordinates already provided by the software to move into the x–y plane accordingly. Subsequently, the extruder moves up into the z-plane to build the next layer until a three-dimensional object is obtained. While depositing these sequential layers, the molten layers of extruded material called the lamina form a bond among the adjacent layers. Likewise, laminates comprising of dissimilar thermoplastic polymers can also be produced using the FDM technique.

The operational flexibility of FDM technology makes it relatively suitable to produce non-conventional laminates as well. The advancements in FDM technology have stimulated its role from just prototyping to a process becoming capable of producing finished products. However, standalone polymer parts produced from FDM technology are mainly used as just prototypes, as they lack strength and durability [8,9,10,11,12]. The mechanical properties of parts produced from FDM have been improved by either introducing reinforced materials into the base filament or by optimizing the printing process parameters. Besides reinforcements, mechanical properties have also been improved with optimized slicing parameters, infill density, and application of appropriate support materials [13,14,15]. Moreover, the mechanical properties of FDM produced parts have also been improved by simultaneously using reinforced filament materials and optimizing the printing process parameters as well. Therefore, by utilizing the 3D printing technique, hybrid composite laminates were produced that have shown better mechanical properties [16,17]. To produce mechanically improved multi-material polymer laminates from FDM technology, the interfacial bond strength (IFBS) and limitations of the printed materials must be studied.

In recent years, very limited studies have been reported that address the mechanical behaviour of additively manufactured laminar composite structures. Zhang used FDM to print and compare IFBS among pristine and reinforced polymers. It was observed that printing orientations and printing parameters coupled with the material composition control the IFBS of the printed samples. As a result, the printing speed of 60 mm/s and layer thickness of 0.18 mm yield the highest shear strengths, which indicate relatively strong IFBS. Moreover, among these composite materials, carbon nanotubes reinforced acrylonitrile butadiene styrene (CNT-ABS) samples printed at 0° orientation possessed a minimum degree of porosity [18]. Singh used the FDM process to 3D print multi-material recycled composite using ABS, polylactic acid (PLA), and high impact polystyrene (HIPA). The tensile tests concluded that the multi-material composite offered better mechanical properties, while varying the laminar sequence of material could induce customization as well [19]. Li compared the interlaminar bonding strength (ILBS) for ABS and polyamide-12 (PA12). The study reported that, in the case of ABS material, the extrusion nozzle temperature did not affect the ultimate tensile strength (UTS). Conversely, for PA12, the extrusion temperature in combination with melt flow viscosity played an important role in achieving better ILBS. It was noted that UTS increased directly with an increase in extrusion temperature up to 250 °C [20]. Patrick studied the interlayer performance of short carbon-fibre reinforced polyamide. The study introduced the infrared preheating system (IPS) to enhance the interlayer tensile strength of the specimen produced through the extrusion process. It was observed that interlayer tensile strength could be characteristic to the interlayer bonding strength [21].

Aliheidari studied the relationship between printing parameters, interlayer structures such as voids, and fracture resistance of FDM parts. It was found that, apart from meso-structural features, printing parameters like extrusion temperature and layer height have a significant effect on fracture behaviour [22]. Kevin Hart printed ABS samples in different orientations to study the relationship between interlaminar fracture properties and lamina orientation angles. It was observed that the energy required for crack propagation across lamina was almost an order of magnitude greater than the energy required for crack propagation along lamina. Therefore, laminar orientation is an important parameter for customization and designing tailored failure behaviours [23]. Islam used FDM technology to introduce reinforcements at an interlaminar level to improve the inter-laminar shear strength of multi directional polymer laminates. A significant improvement of 28% in inter-laminar shear strength was observed. Consequently, it helped in minimizing the delamination in multi-directional laminates [24]. Yin studied the effect of printing process parameters on the IFBS of thermoplastic polyurethane (TPU)/ABS bi-material structures. The study concluded that building stage temperature was the most effective parameter among the considered parameters. IFBS was increased from 0.86 to 1.66 MPa when the building stage temperature was adjusted from 30 to 68 °C [25]. Tamburrino studied material printing order, slicing pattern, and degree of infill density as the considered parameters to measure the adhesion strength of the three different filaments. It was concluded that, apart from thermodynamic diffusion mechanisms, mechanical interlocking strategies are also effective in increasing IFBS [13].

The above-presented literature demonstrates that the interfacial bonding strength (IFBS) of the FDM parts can be characteristic to the printing parameters. However, these interfacial properties are likely to change with material as well. The available literature presents studies regarding the interlaminar bonding strength of FDM printed parts for mono-material filaments or reinforced polymer filaments only. Additionally, multi-material hybrid composites offer relatively improved mechanical properties and have more applications than mono-material printed parts. The FDM printed hybrid laminar composite comprising of carbon fiber reinforced polylactic acid and ABS (CF-PLA/ABS) has yielded better mechanical performance than standalone CF-PLA or ABS printed parts [16,26]. Until now, to the best of the author’s knowledge, no study in the literature presents research on the bonding strength at the interface of the CF-PLA/ABS composite as a function of printing parameters. Therefore, there is a need to systematically study the relationship between printing parameters and bonding strength at the interface of the hybrid composite of CF-PLA and ABS. Moreover, the prediction model from the current study can be used to simultaneously predict and customize the IFBS by selecting the appropriate values for the considered printing parameters. This work is an extension of previously done research that has established the capability of FDM technology for producing hybrid composite laminate parts that bear higher mechanical properties as compared with their parent materials [16].

## 2. Materials and Methods

In this study, a hybrid laminar composite was printed by employing two materials, namely ABS and CF-PLA, as the parent materials. The ABS filament was supplied by Xplorer3D (UAE), having filament density of 1.07 g/m^3^, and the recommended extrusion and bed temperature were 220 °C and 90 °C, respectively, as also recommended in the literature [27]. CF-PLA filament was supplied by MatterHackers (Lake Forest, CA, USA), having 10% carbon fibres by mass. The carbon fibres were sufficiently small enough to easily pass through the extrusion nozzle, and long enough to provide additional strength to PLA matrix. The extrusion and bed temperature of around 210 °C and 80 °C, respectively, were maintained during the printing process. The diameters of both filaments were 1.75 mm, which were compatible with the extrusion nozzle of the Xplorer3D printer (Xplorer 3D, Dubai, UAE).

**Figure 1:**
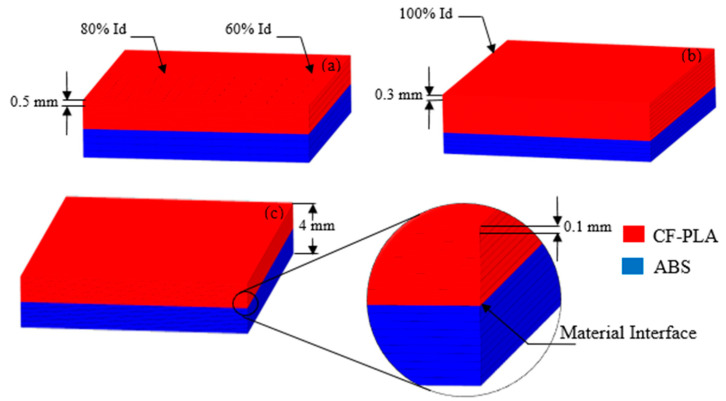
(**a**) Layer thickness ratio of 1.00; (**b**) layer thickness ratio of 0.63; (**c**) layer thickness ratio of 0.25. CF-PLA, carbon fiber reinforced polylactic acid; ABS, acrylonitrile butadiene styrene.

The scope of this study covers the effect of four printing parameters, namely, printing speed (S), infill density (ID), layer thickness ratio (LTR), and layer height (LH), on the interfacial bond strength (IFBS) of the hybrid laminar composite produced through the FDM process. Printing speed is attributed as the speed of the nozzle with which it travels over the printing bed during the printing process. Infill density is the total amount of printing material inside the periphery of the printed part, which is usually measured in percentage. Layer height is the thickness of the layer when the filament is extruded from the printing nozzle. Layer thickness ratio is the composition ratio of two different materials (ABS and CF-PLA) in the single composite sheet as different LTRs constitute a different percentage of individual parent materials to form a composite laminate, as illustrated in Figure 1. Therefore, LTR was varied at three different levels of 0.25, 0.63, and 1. Table 1 shows the amount of individual parent material in the composite for each level of LTR.

A statistical approach called the design of experiment was adopted to design the set of experiments for testing as well as to analyze the results. This statistical method requires a relatively fewer number of experiments to explore the effects of various input variables on the target variables or responses. For an investigation of the output variable, interfacial bond strength, in this case, response surface methodology (RSM) was employed in design expert (DX12) software (Stat-Ease, Minneapolis, MN, USA). RSM computes the individual and combined effects with a relatively lower number of experiments and yields good statistical accuracy as well as trends [28,29]. The lower and upper levels of the considered printing parameters were selected according to the preceding research work [16], also given in Table 2. The experimental plan is presented in Table 3, which constitutes 22 experiments having four replicates, while each of the four parameters was varied over three levels.

The test specimens were printed by employing the values of printing parameters for each run, as shown in the experimental plan (Table 3). The printing of specimen was carried out by Xplorer3D, while the nozzle diameter, nozzle temperature, and bed temperature were kept constant at 0.75 mm, 210 °C, and 90 °C, respectively. The printing pattern specifically called the raster angle was kept as the 0/90° pattern, as it offers more strength than the 45/45° printing pattern [30]. The geometry of the specimen to conduct IFBS is a square of 19 mm × 19 mm, having a total thickness of 4 mm. To produce the composite bi-material sample, firstly, ABS material was used to print laminate on the printing bed until the pre-defined thickness was achieved. It was then instantly followed by the printing of CF-PLA filament onto the already printed ABS laminate in a single printing operation, which resulted in the fusion of two materials at the interface, hence forming an interfacial bond. The transition of printing filament from ABS to CF-PLA was governed by the combination of layer thickness ratio and layer height, as given in Table 4.

## 3. Results and Discussion

### 3.1. Interfacial Behavior under Uniaxial Tensile Loading

**Figure 2:**
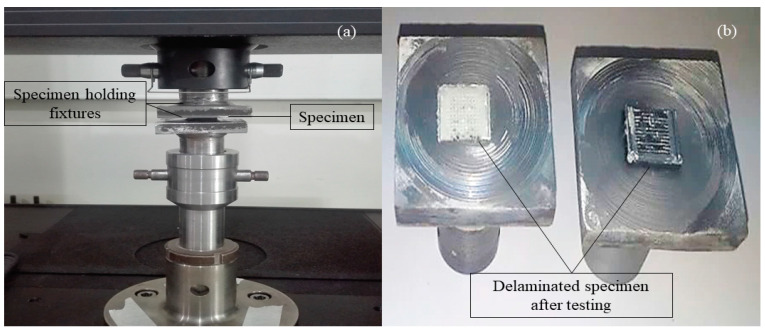
(**a**) Pre-testing sample; (**b**) post-testing sample.

**Figure 3:**
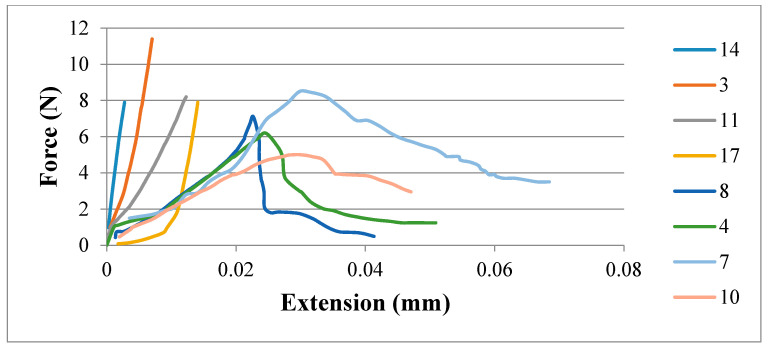
Force vs. extension graph for representative specimens.

Tests were conducted to measure IFBS of the specimens using an ultimate tensile machine (UTM) having a capacity of 30 kN load with a constant strain rate of 2 mm/min [13,25]. Table 3 presents the results for the bond strength of hybrid composite sheets. Epoxy was applied on both sides of the specimens to adhere them with the testing fixtures to perform tests. Figure 2 represents the sample being tested on the UTM machine as well as the post-testing sample. The results of IFBS testing are presented in Table 3, which shows that the maximum bond strength of 20.5 MPa was achieved for specimen number 12, while the minimum value of bond strength was recorded to be 0.56 MPa for specimen number 19. Figure 3 shows the graph of force versus extension for IFBS of the representative specimens. It indicates two failure modes, which are failure mode 1 and failure mode 2, as also mentioned in Table 3. In Figure 2, specimen number 3, 7, 11, and 14 represent failure mode 1, whereas specimen number 4, 7, 8, and 10 represent failure mode 2.

**Figure 4:**
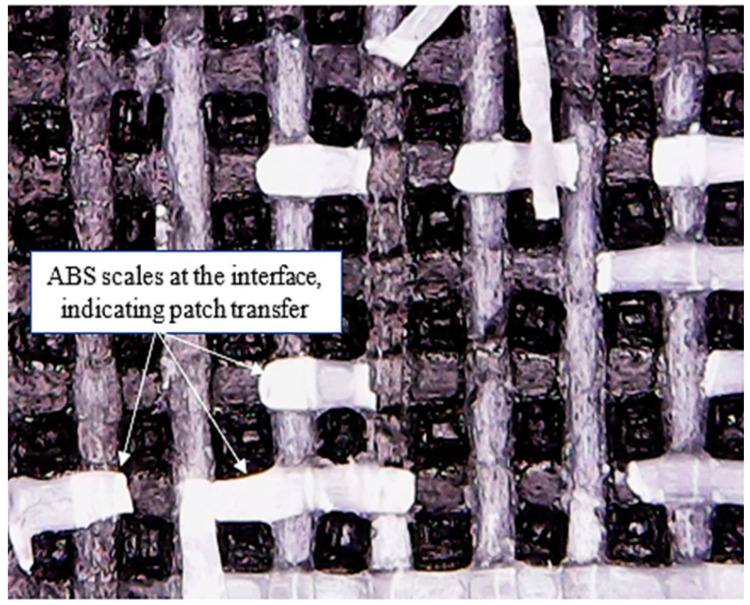
Patch transfer of ABS lamina on the CF-PLA portion after delamination.

**Figure 5:**
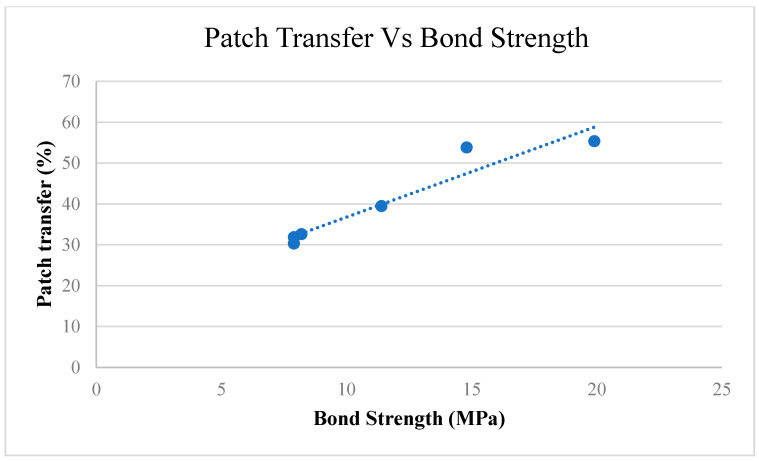
Patch transfer vs. bond strength.

In the first type of failure (failure mode 1), catastrophic failure behaviour is observed during the delamination process. It was statistically observed that failure mode 1 has an average bond strength of 9.63 MPa, whereas theaverage bond strength for failure mode 2 was found to be 4.36 MPa. Therefore, it can be deduced that specimens failing under failure mode 1 have a relatively higher bond strength than specimens failing under failure mode 2. Moreover, visual inspection through an optical microscope of the interfacial surface of tested specimens revealed that failure mode 1 exhibits patch transfer behaviour as well. In patch transfer behaviour, one of the laminae from the two dissimilar materials at the composite interface remains attached to the other one after being subjected to delamination forces at the laminar interface. As evident from Figure 4, laminae of ABS material have remained bonded with CF-PLA at the interface of the ABS/CF-PLA composite even after being subjected to delamination. It refers to a strong inter-laminar bond that occurs at the interface of the composite, which could be a result of favourable printing parameters. This patch transfer behaviour (scales of ABS material on CF-PLA portion) indicates the strong bond developed between the two parent materials of the composite at the interface. An in-depth investigation of the specimens revealed a direct relationship between the percentage of patch transfer area versus bond strength, as shown in Figure 5. Therefore, for specimens failing under failure mode 1, interlaminar bond strength is most likely governed by the percentage of patch transfer area.

**Figure 6:**
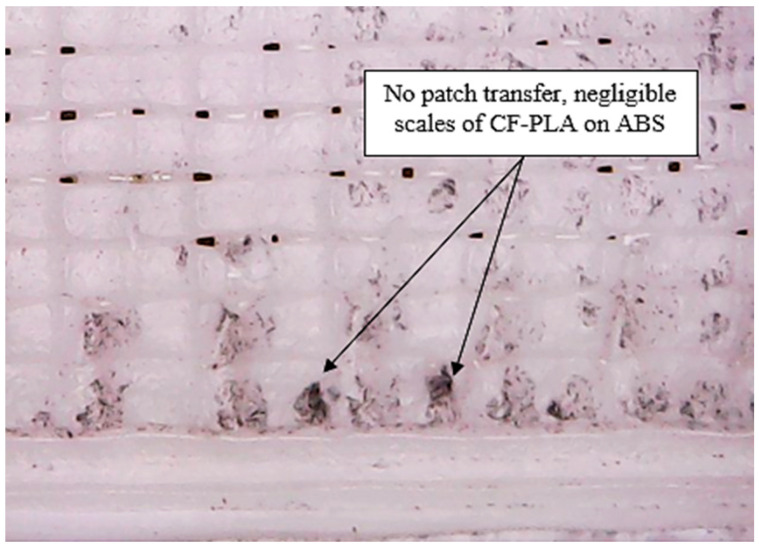
No patch transfer from the CF-PLA to ABS portion after delamination.

In the case of failure mode 2, in contrast with failure mode 1, no catastrophic behaviour is observed. The relatively large values of extension for failure mode 2 infer that these specimens do not possess strong inter-laminar bonding when compared with failure mode 1. This could be due to the unfavourable printing parameters incorporated during the specimen printing. Furthermore, Figure 6 presents that no patch transfer has occurred after the delamination process, and the ABS laminae remain intact to their laminar mesh. At the same time, no beads or fibres of CF-PLA are found attached to this portion of the composite. Therefore, this supports and corroborates the finding that there exists a weak interfacial bonding strength for specimens failing under mode 2. Therefore, patch transfer is fair evidence that indicates interfacial bonding is related to the failure modes. To conclude, two types of failures occurred: (1) Patch transfer: This infers that, for corresponding printing parameters, intra layer bonding of the materials was weaker than the interfacial bonding between the two laminates. Resultantly, the patch of material with weaker intra layer bond transferred from one laminate to other laminate during the pull test. (2) No patch transfer: This indicates that intra layer bonding of the materials was stronger than the interfacial bonding between the two laminates. Therefore, no patch transfer (from one laminate to other laminate) was observed and, resultantly, the two laminates decoupled. In other words, we can also say that mode 1 failure occurs when the inter-layer bond of the laminates is weaker than the inter-laminates bond. Meanwhile, mode 2 failure occurs when the inter-laminates bond is weaker than the inter-layer bond of the laminates.

### 3.2. Analysis of Variance (ANOVA)

To investigate the significant printing parameters that yield strong IFBS, analysis of variance (ANOVA) was conducted. The ANOVA for bond strength is presented in Table 5. Four parameters, namely, printing speed (S), infill density (ID), layer height (LH), and layer thickness ratio (LTR), were considered in the current study, while interfacial bond strength (IFBS) was the response variable against the given printing parameters. A parameter was considered to have a significant effect if its *p*-value was ≤0.05, which employs a confidence level of more than 95%. As can be seen from Table 5, the model is significant, which means the quadratic model used for the ANOVA response surface is statistically correct. Further, following the predefined criterion, printing speed and layer height are the significant individual parameters. It was observed from Table 5 that, among the considered printing parameters, most of them interact with each other. This infers that the nature of the influence of the considered parameters is associated with the interacting ones. These interactions refer to the fact that a combined effect on the response variable was posed when the effect of one parameter is dependent on its corresponding companion parameter. The lack of fit, in this case, has a *p*-value > 0.1, which indicates towards its insignificance; therefore, the model can successfully interpolate between two design points. The analysis also revealed that the standard deviation for the response was 0.72, which shows that dispersion was low, and the results possess good repeatability.

### 3.3. Effect of Printing Parameters on Interfacial Bond Strength (IFBS)

**Figure 7:**
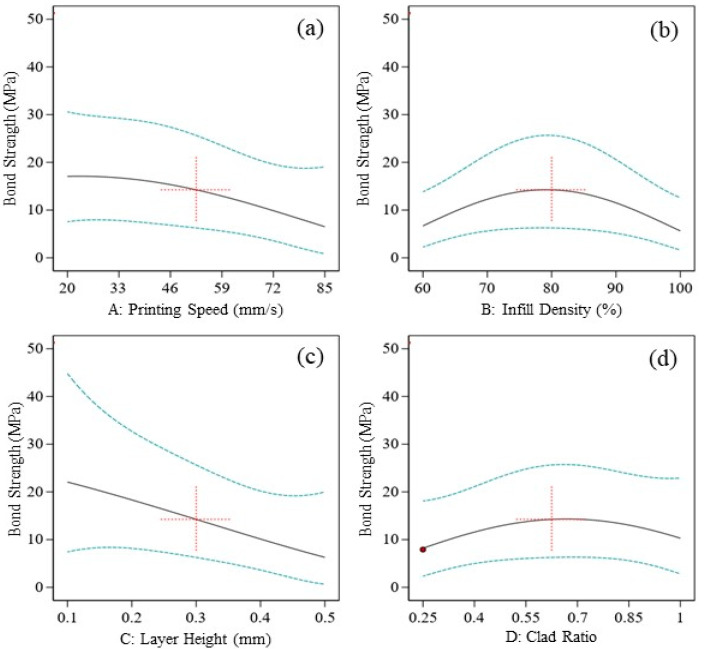
Individual effects of (**a**) printing speed, (**b**) infill density, (**c**) layer height, and (**d**) layer thickness ratio on bond strength.

Generally, the effect of input parameters on the output response can be studied with the help of single parameter versus response graphs, as shown in Figure 7a–d. However, when the two-factor interaction (2FI) model is used for ANOVA, it becomes imperative to study interactive graphs or 3D hypersurfaces as well. In this study, Figure 7a–d presents the individual effect of printing parameters on bond strength. It is evident from Figure 7b,d that infill density and layer thickness ratio have a negligible or no effect on bond strength, which is also supported from Table 5, where both parameters were marked as insignificant parameters. Conversely, printing speed and layer height were considered as significant parameters. From Figure 7a, it was noted that bond strength displayed an inverse relationship with printing speed. It is generally observed that, at high printing speed as the raster is still in semi-molten phase, there is a likelihood of improved IFBS due to better interlayer fusion. However, a high printing speed induces distortions as well prevents good IFBS. Therefore, there exist competing mechanisms regarding the relationship of printing speed with IFBS, hence it varies from material to material. In this case, a high printing speed did not allow the printing beads to settle down properly; thus, the latter effect was found to be a dominant mechanism. Additionally, a high printing speed lacks printing accuracy and creates distortions in the printing patterns. Furthermore, distortions were also responsible for the poor quality of bonded layers, which could potentially be due to non-uniform temperature gradient between the consecutive layers [31]. It also requires the feedstock to be flowable enough at the allowable printing temperature to flow out of the extrusion nozzle at the respective speed to deal with the high printing speed. Because of the reasons mentioned above, the subsequent upcoming layer of different material in the transition phase cannot properly fuse to the previously printed layer. Consequently, the interfacial bond strength of 3D printed composite materials is compromised. Therefore, in this case, the high printing speed is unsupportive for printing thermoplastic materials. On the other hand, the low printing speed supplements the interlayer fusion by allowing the subsequent layer to adhere properly, hence better bonding strength is achieved. The other significant printing parameter that individually affects the bond strength is layer height. Its behaviour towards bond strength is very synonymous to the printing speed, as shown in Figure 7c. As the layer height increases, bonding strength starts decreasing gradually. It may be because the increased layer height incorporates air gaps into the printing pattern, which means that there exists unwanted porosity in the specimen. The air gaps due to increased layer height will not allow the rasters to properly adhere and fuse with each other. Therefore, a weak interfacial bonding will occur due to increased layer height. Contrary to this, low values of layer height will incorporate less volume of air gaps, thus there will be a greater likelihood of strong interfacial bonding strength.

**Figure 8:**
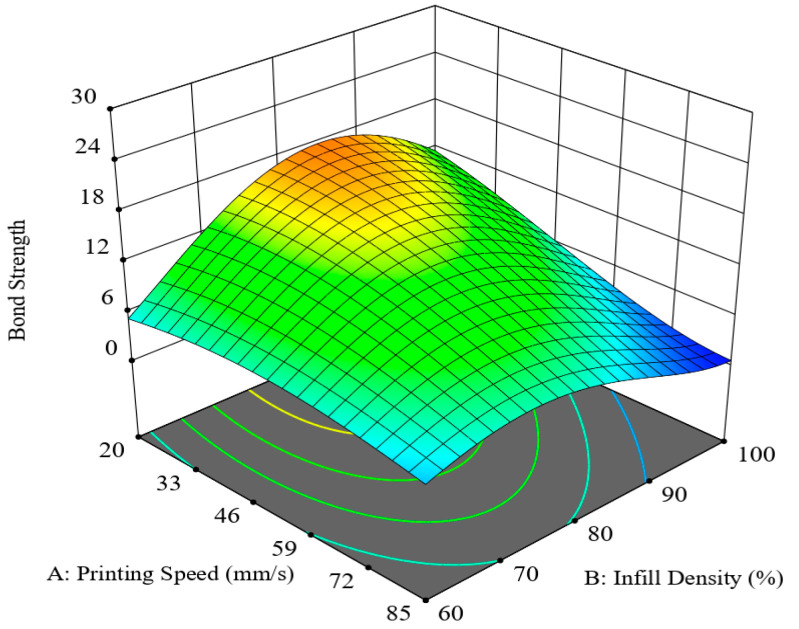
Combined effect of printing speed and infill density (S-ID hypersurface).

**Figure 9:**
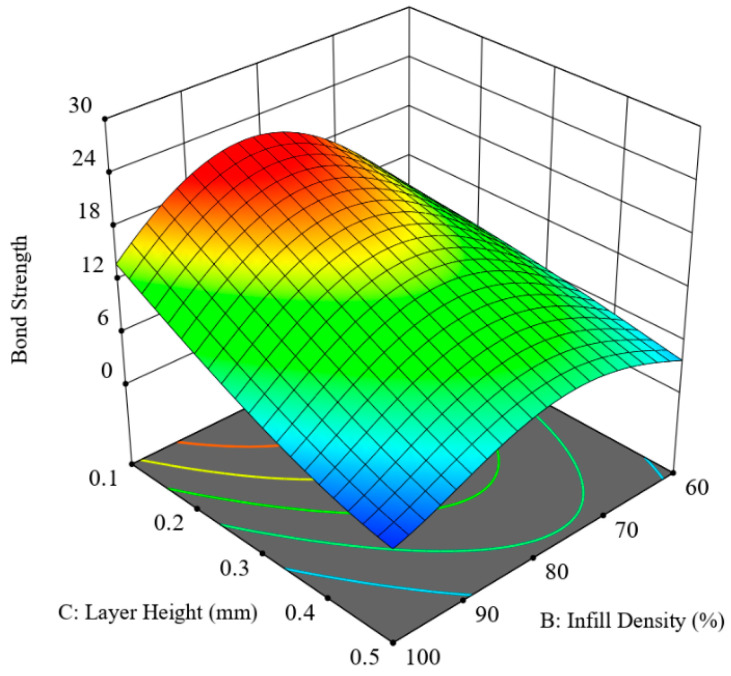
Combined effect of layer height and infill density (LH-ID hypersurface).

Figure 8 shows the synergic effect of printing speed and infill density in an S-Id hypersurface with two other parameters, layer height and layer thickness ratio, fixed at 0.3 mm and 0.625, respectively. The interactive graph of the two companion parameters reveals that infill density of about 85% and a low printing speed of 20 mm/s are the most favourable printing conditions to achieve high bonding strength. Conversely, maximum infill density coupled with maximum printing speed yielded the lowest values of bonding strength. It could be because, for 100% infill density (no air gap), there is a likelihood of developing stress accumulation by restricting heat transfer, thus the printed part needs a longer time to dissipate heat [31]. If the minimal air gap is present in the printing pattern, there would be some ventilating space available for efficient heat transfer. That is why 100% infill density and 80 mm/s printing speed produced lower bonding strength. It was also noted that, for the Lh-Id hypersurface for the interaction of layer height and infill density with the rest of the parameters set at average settings, a similar behaviour occurred, as can be seen from Figure 9.

**Figure 10:**
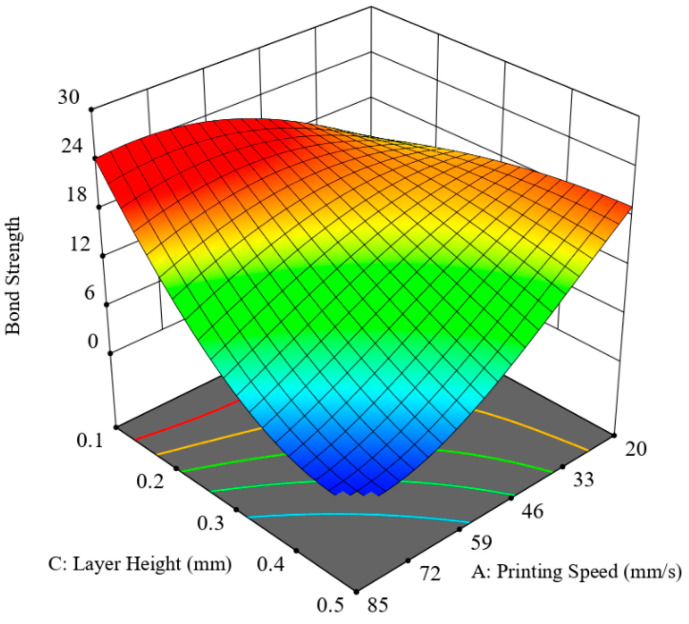
Combined effect of layer height and printing speed (LH-S hypersurface).

Figure 10 presents the collective influence of layer height and printing speed with the other parameters, infill density and layer thickness ratio, fixed at 80% and 0.625, respectively. It has been revealed that, as soon as the values of layer height and printing speed are increased, a consistent decline in bonding strength occurs. Therefore, it can be deduced that a combination of high layer height and increased printing speed exacerbate the IFBS and the two considered parameters are the most incompatible interactions. The reason being, as already discussed for individual effects of these printing parameters, higher values yielded poor bond strength. Therefore, it becomes evident that superimposing their high values would produce catastrophic results for bond strength. However, an interesting trend has revealed that a low value for one of the two considered printing parameters would yield good bond strength, irrespective of the other parameter. Furthermore, the maximum achieved bond strength value of 20.5 MPa becomes apparent when both printing parameters were at their low extremes. Therefore, it can be said that both printing parameters complement each other to produce high interfacial bond strength.

**Figure 11:**
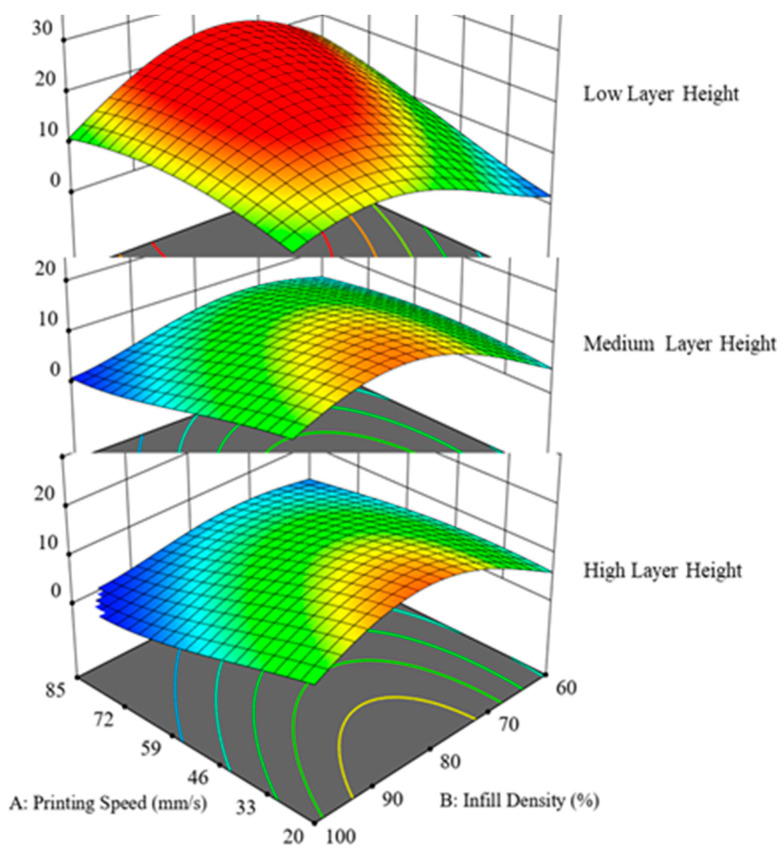
Effect of layer height superimposed on the combined effect of printing speed and infill density.

**Figure 12:**
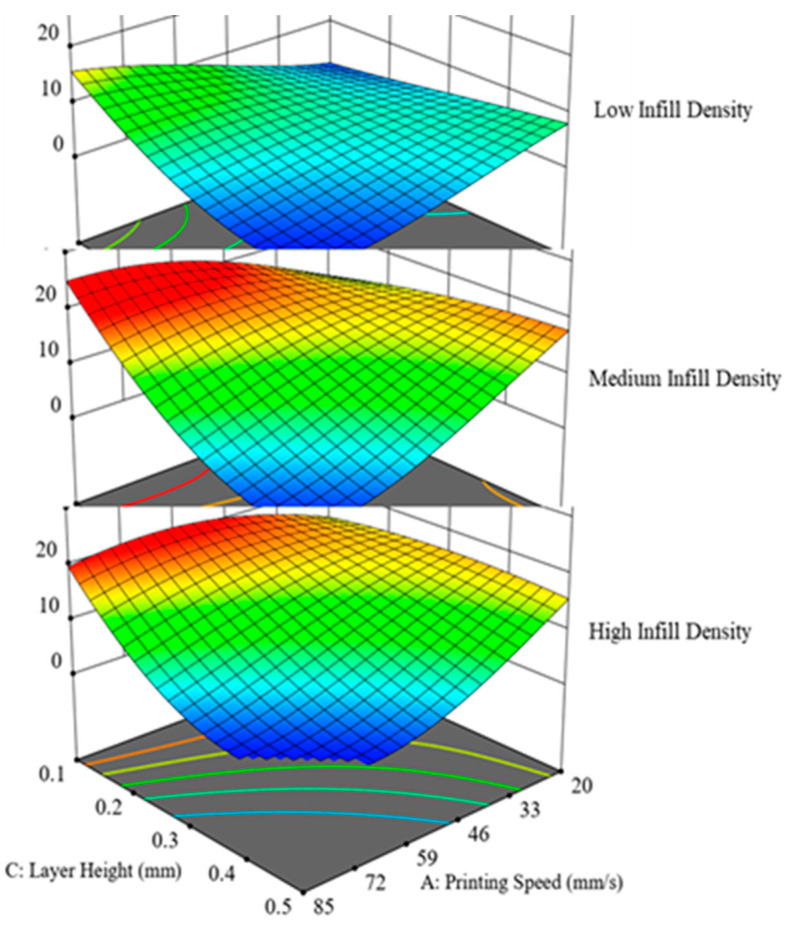
Effect of infill density superimposed on the combined effect of layer height and printing speed.

To further investigate and explore the behavior of IFBS with the changing printing parameters, superimposed effects of selected printing parameters on IFBS were studied. Figure 11 shows the superimposed effects when printing speed and infill density were coupled with varying layer height. To explore the superimposed effects, layer height was varied at 0.1, 0.3, and 0.5 mm, respectively. Keeping the layer height at a low setting (0.1 mm) while considering the printing speed and infill density, the maximum bonding strength can be achieved regardless of whether the layer thickness ratio is high or low. However, it was observed that, by increasing the value of layer height, the bonding strength immediately started decreasing. Figure 11 corroborates this trend as the S-Id hypersurfaces took a shift towards lower values of bonding strength when layer height values of 0.3 mm and 0.5 mm were employed. Additionally, it was also noted that IFBS was constrained to a maximum of 10 MPa using 0.5 mm as layer height. Therefore, it was noted that such unfavourable printing combinations posed a diminishing effect on IFBS and, consequently, its magnitude was reduced to half of the achievable value. Likewise, Figure 12 presents the superimposed effects of infill density on printing speed-layer height (S-LH) hypersurface. Infill density was employed at 60%, 80%, and 100% settings. It is evident from Figure 11 that a low infill density was detrimental for the IFBS and reduced to as low as 13 MPa, whereas the settings with an infill density of 80% and 100% improved the bonding strength. From the above discussion, it can be concluded that the considered superimposed printing conditions only affect the magnitude of IFBS, whereas the nature of the response remains unchanged.

### 3.4. Prediction Model and Optimum Printing Conditions

The combined effects of printing parameters on the bond strength can be represented into one hypersurface known as the empirical model. For the current study, the empirical model is given below:

(1) Sqrt (Bond Strength)=3.84−0.89A−1.14C−0.75AB−1.60AC+0.54C

**Figure 13:**
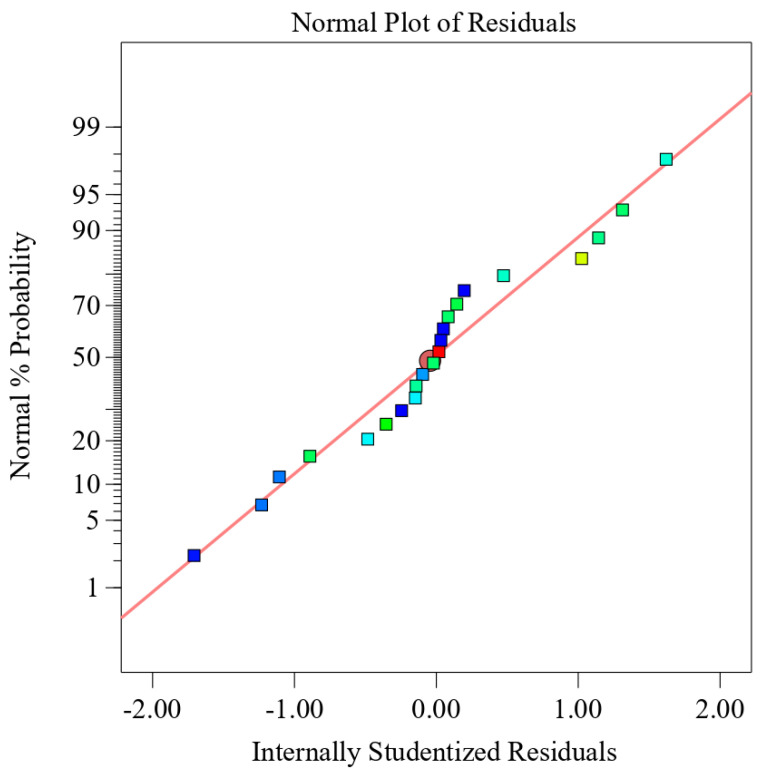
Normal probability plot.

Generally, two criterions can be used to measure the fitness of the empirical model: *R*^2^ value and normal distribution. The determination coefficient (*R*^2^) for bond strength was 0.9021, which is close to 1. This infers that 90.21% of the total variation in bond strength can be derived by the empirical models developed in the experimental design, which represents that the relationship between the experimental and estimated results is in good agreement. The signal to noise ratio is measured with adequate precision, while a ratio of greater than 4 is desirable. For the current regression model, a value of 8.36 indicates a fair signal, which follows that the present model can be used to navigate the design space. Figure 13 represents the normal distribution of internally studentized residuals, which shows that residuals follow the normal distribution. Therefore, these tests validate that the model is fairly accurate.

It has been established that printing parameters affect the IFBS of the printed specimen. Therefore, optimum printing conditions were determined to achieve suitable IFBS. The optimization performed by the software was an iterative process, and a total of 100 solutions were obtained. The optimization was achieved by employing the desirability function as described in [32] and given in Equation (2).

(2) D=(d1r1·d2r2………·dmrm)1(r1+r2+…+rm)

where *d_i_* is the desirability of an individual response, *r* is the weightage of each response, and *D* is the collective desirability of considered responses. Meanwhile, the objective was to maximize the bond strength. For optimization in the current study, a single solution was accepted, having the highest desirability function of 0.89. The printing speed of 50.54 mm/s, infill density of 79.82%, layer height of 0.15 mm, and layer thickness ratio of 0.49 were found to be the best printing parameters.

## 4. Conclusions

The current study investigated the interfacial bond strength (IFBS) of multi-material laminar composites manufactured through the fused deposition modelling process. The research aimed to study the influence of printing parameters on the IFBS by employing response surface methodology. Following are the important findings of the current study:

1. Optical microscopy revealed two types of failure modes after the specimens were subjected to uniaxial tensile loading. Mode 1 exhibited patch transfer behavior that indicated a strong interfacial bond between the two constituent materials (ABS and CF-PLA). Whereas, in case of failure mode 2, no scales or patch transfer of laminae material were found, which was characteristic to a weak interfacial bond. Moreover, a linear relationship between patch transfer percentage and IFBS was discovered.
2. Printing parameters were found to be very effective in determining the IFBS of the composite laminates. The ANOVA suggested that low printing speed and low layer height coupled with high infill density yield better IFBS. Moreover, the superimposed effects revealed that the magnitude of IFBS varies with varying superimposed parameters; however, the nature of IFBS remains the same.
3. The empirical relation devised would guide the researchers to successfully predict the IFBS for any given printing parameters. Meanwhile, optimum printing parameters yielding good IFBS were found to be printing speed of 50.54 mm/s, infill density of 79.82%, layer height of 0.15, and layer thickness ratio of 0.49.

## Figures and Tables

**Table 1:** Layer thickness ratio vs. material percentage. CF-PLA, carbon fiber reinforced polylactic acid; ABS, acrylonitrile butadiene styrene.

Layer Thickness Ratio	Material Percentage by Volume	
1.00	50% CF-PLA	50% ABS
0.63	63% CF-PLA	37% ABS
0.25	75% CF-PLA	25% ABS

**Table 2:** Range of parameters.

Factor	Symbol	Unit	Low Level	Mid-Level	Upper Level
Printing speed	S	mm/s	20.00	52.50	80.00
Infill density	ID	%	60.00	80.00	100.00
Layer height	LH	mm	0.10	0.30	0.50
Layer Thickness ratio	LTR	-	0.25	0.63	1.00

**Table 3:** Experimental plan and test results.

Specimen No.	Printing Speed (mm/s)	Infill Density (%)	Layer Height (mm)	Layer Thickness Ratio No Units	Interfacial Bond Strength (MPa)	Failure Mode
	S	ID	LH	LTR	IFBS	1/2
1	52.5	100	0.5	1	2.5	2
2	20	60	0.1	0.25	4.2	2
3	80	100	0.1	0.25	11.4	1
4	52.5	100	0.3	0.63	6.2	2
5	52.5	80	0.3	0.25	7.92	1
6	20	100	0.5	0.25	6	1
7	20	60	0.5	0.63	8.5	2
8	80	80	0.3	0.63	7.1	2
9	52.5	60	0.1	1	2.3	2
10	52.5	60	0.3	0.63	5	2
11	80	60	0.5	0.25	8.2	1
12	20	80	0.1	1	20.5	1
13	80	100	0.1	1	3.1	2
14	52.5	60	0.1	1	7.9	1
15	52.5	60	0.5	0.25	6.3	1
16	80	60	0.5	1	3.4	2
17	20	100	0.1	0.25	7.9	1
18	20	60	0.3	1	2.43	1
19	20	100	0.5	0.25	0.56	2
20	80	60	0.1	0.25	14.8	1
21	20	100	0.1	1	4.9	1
22	20	60	0.3	1	7.4	1

**Table 4:** Material transition against various printing parameters.

Experiment No.	Layer Thickness Ratio	Layer Height	Total Number of Layers	Layers of ABS	Layers of CF-PLA
1	1.00	0.50	8.00	4.00	4.00
2	0.25	0.10	40.00	10.00	30.00
4	0.63	0.30	14.00	9.00	5.00

**Table 5:** Analysis of variance (ANOVA) for bond strength.

Source	*p*-Value	Significance (Y/N)
Model	0.0249	Y
A—Printing Speed	0.0317	Y
B—Infill Density	0.5621	N
C—Layer Height	0.0036	Y
D—Layer Thickness Ratio	0.3364	N
AB	0.0394	Y
AC	0.0072	Y
AD	0.4049	N
BC	0.0809	N
BD	0.2369	N
CD	0.0722	N
A^2^	0.3571	N
B^2^	0.0209	Y
C^2^	0.8441	N
D^2^	0.2110	N
Lack of Fit	0.9692	N
